# Aberrant Resting-State Cerebellar-Cerebral Functional Connectivity in Methamphetamine-Dependent Individuals After Six Months Abstinence

**DOI:** 10.3389/fpsyt.2020.00191

**Published:** 2020-03-31

**Authors:** Xiaotong Li, Hang Su, Na Zhong, Tianzhen Chen, Jiang Du, Ke Xiao, Ding Xu, Weidong Song, Haifeng Jiang, Min Zhao

**Affiliations:** ^1^Shanghai Mental Health Center, Shanghai Jiao Tong University School of Medicine, Shanghai, China; ^2^Department of Physiological Rehabilitation, Shanghai Drug Rehabilitation Administration Bureau, Shanghai, China; ^3^Shanghai Key Laboratory of Psychotic Disorders, Shanghai Mental Health Center, Shanghai, China; ^4^Institute of Psychological and Behavioral Science, Shanghai Jiao Tong University, Shanghai, China; ^5^CAS Center for Excellence in Brain Science and Intelligence Technology (CEBSIT), Chinese Academy of Sciences, Shanghai, China

**Keywords:** addiction, cerebellum, methamphetamine, functional connectivity, default-mode network

## Abstract

**Background:**

Structural and functional alterations in the cerebellum have been consistently reported in addiction literatures. However, evidence implicating the resting-state cerebellar-cerebral functional connectivity in methamphetamine (MA) use disorder still remains limited.

**Methods:**

Resting-state functional magnetic resonance imaging (fMRI) scans were obtained from 34 MA dependent individuals with about six months abstinence and 31 healthy controls (well matched for age, gender and education) in this study. Seed-based functional connectivity analysis was employed to investigate the differences in cerebellar-cerebral functional connectivity between two groups. The correlations between significant functional connectivity and each clinical characteristic were also explored.

**Results:**

Compared to healthy controls, MA dependent individuals showed disrupted functional connectivity between the cerebellum and several cerebral functional networks, including the default-mode, affective-limbic, and sensorimotor networks. Within the MA group, functional connectivity of the right cerebellar lobule VI-precuneus coupling was negatively correlated with addiction severity.

**Conclusion:**

The present study suggests that cerebellar dysfunction, in particular aberrant cerebellar-cerebral functional connectivity, might involve in neurobiological mechanism of MA dependence, which supply a potential target for therapeutic interventions in the future.

## Introduction

Methamphetamine (MA) use disorder is a severe and chronic mental disorder, characterized by compulsive drug seeking and taking (1). The prevalence of MA use is continuously increasing and ranked second in illegal drug use worldwide (2). As a kind of amphetamine-type stimulant (ATS), MA has always been of particular concern for its strong neurotoxicity, high risk of relapse, and comorbidity with psychotic disorders (3, 4).

Substantial scientific researches have demonstrated the impact of MA use on neuropsychological functions (5, 6). Long-term MA exposure is always linked to a broad range of impaired functional domains, including executive control, emotional regulation, reward processing, and goal-directed behaviors (7). In addition, altered activations in distributed brain areas, such as prefrontal cortex, striatum and hippocampus, have been reported to involve in cognitive and emotional impairments in MA users (8, 9). As the large-scale brain network model has been proposed, rather than isolated brain regions, recent literatures have emphasized several intrinsically defined networks in the pathophysiology of addiction, like the executive control network (ECN), affective-limbic network, default-mode network (DMN), etc. (10) The DMN, anchored in medial prefrontal cortex (mPFC) and posterior cingulate cortex (PCC), is reported to be responsible for compromised cognitive function and obsessive rumination about drugs in addictive disorders (11–13). The affective-limbic network, covering a collection of subcortical structures such as amygdala, thalamus and cingulate gyrus, is engaged in dysregulated emotional activities, drug-related cue induced conditioning learning and memory (13, 14). Moreover, clinical observations have revealed altered activation patterns of these large-scale networks accompanied by reduction in clinical features when drug users remained abstinent (8).

Notwithstanding the prominent progress in addiction field, there has been little attention paid to the role of the cerebellum in neurobiological mechanism of drug addiction. The cerebellum is traditionally considered as one of the core structures underlying motor function, but increasing evidence has demonstrated that it is also implicated in brain processes associated with cognitive control, affective regulation, learning and memory which are impaired domains in addictive behaviors (15–17). Furthermore, functional neuroimaging researches have shown that the cerebellum has reciprocal loops with many cerebral functional networks, including the aforementioned ECN, DMN and affective-limbic network (18, 19). Animal studies have consistently reported that there are widespread projections connecting cerebellum with the cortico-striatal-limbic circuitry where addictive drugs can act and induce the change of synaptic plasticity (20, 21). Notably, a convergence of clinical investigations has identified the structural changes in cerebellum after long-term exposure to heroin, alcohol and psychostimulants including MA (22, 23). As compared to healthy controls, the MA dependent individuals exhibited reduced volume in the cerebellar gray matter and gained greater volume while maintained abstinence for six months (23). On the other hand, numerous post-modern studies have revealed the co-activations in cerebellum with a number of cortical and subcortical areas such as orbitofrontal cortex, hippocampus and cingulate gyrus, when presenting the drug-related cues (22, 24, 25). However, evidence implicating the resting-state cerebellar-cerebral functional connectivity (FC) in addiction still remains limited. Whether there are abnormalities in the cerebellar-cerebral functional networks, especially intertwined in substance use disorders in every stage of addiction cycle, is an important knowledge gap that might be addressed.

In view of the common structural and functional impairments in cerebellum and large-scale networks in substance dependent individuals (22, 25), the present study examined resting-state cerebellar-cerebral FC in long-term abstinent MA dependent individuals with matched healthy controls for a comprehensive investigation of executive control, default-mode, affective-limbic and sensorimotor networks, including seeds in cerebellum involved in corresponding function (19, 26). We hypothesized that there would be disrupted FC in cerebellar-cerebral pathway that may subserve the dysregulation in executive control, emotional processing and sensorimotor function in MA dependent individuals with long-term abstinence, and that these altered circuits may be correlated with clinical characteristics, such as the addiction severity.

## Materials and Methods

### Participants

For this study, thirty-seven MA dependent individuals who underwent a long-term residential MA-addiction treatment were recruited from Shanghai drug rehabilitation center. All 37 MA patients met the DSM-5 criteria for MA use disorder (MUD). The exclusion criteria for the MA group were: (1) other substance dependence except nicotine within the past five years; (2) other axis I psychiatric disorders comorbidity including schizophrenia, depression, bipolar disorder, etc.; (3) any history of brain diseases such as head injury, head surgery and neurological diseases; (4) serious physical illness; (5) Magnetic Resonance Imaging (MRI) contraindications. In order to control the potential effects, all of the MA dependent subjects received no medication and maintained abstinence before their MRI scans.

Thirty-three healthy controls (HCs) were recruited from the community, and well matched for age, gender and education. All HCs were screened for psychiatric disorders and medical problems by two experienced psychiatrists. None of them had a diagnosis of psychiatric disorder, any history of drug use or brain diseases.

The study was approved by the ethics committee of Shanghai Mental Health Center. All participants were informed of the procedure and provided written informed consent.

### Clinical Assessment

Each subject was interviewed and completed a structured questionnaire which collected social-demographic information (age, gender, level of education, etc.) and drug-use history (age of first MA-use, duration of MA use, daily dose per day, etc.). MA craving was assessed using a Visual Analogue Scale (VAS) (27). For an additional measurement of past 30-days substance use before they entered the treatment, we administered the Addiction Severity Index (ASI) for MA group (28, 29). Then, for each MA dependent subject, we calculated the total score of ASI for addiction severity and the score of drug-use dimension for drug use severity (28, 29).

### MRI Data Acquisition

Imaging scans were acquired on a 3T Siemens Trio scanner (Erlangen, Germany) with a 32-channel head coil. Participants were instructed to relax and remain still with their eyes closed but remain awake, and foam pads were used to minimize head motion. A high-resolution T1-weighted 3D anatomical image was collected by a MPRAGE sequence with the following parameters: repetition time (TR) = 2300 ms, echo time (TE) = 3 ms, flip angle = 9°, field of view (FOV) = 256 mm, matrix size = 256 x 256, 192 slices, voxel size = 1 x 1 x 1 mm^3^. The resting-state functional images were collected with the following parameters: TR = 2000 ms, TE = 30 ms, flip angle = 62°, FOV = 240 mm, matrix size = 120 x120, voxel size = 2 x 2 x 2 mm^3^, 72 slices, slice thickness = 2 mm, no intersection gap, each functional run lasted for 8 minutes and contained 240 volumes.

### Data Processing

Imaging data were analyzed using a toolbox for Data Processing & Analysis for Brain Imaging (DPABI_v2.3) (30) which based on statistical parametric mapping program (SPM12) (http://www.fil.ion.ucl.ac.uk/spm/software/spm12) and Resting-State fMRI Data Analysis Toolkit (REST) (http://restfmri.net/forum/REST_V1.8). For each participant, the first 10 volumes were discarded for signal equilibrium and subject familiarization, and the remaining 230 consecutive volumes were slice timing corrected and realigned for head motion correction. The individual structural images were co-registered to the mean functional images and segmented into cerebrospinal fluid, gray matter, and white matter. Then several nuisance covariates, including six head motion parameters, signal extracted from cerebrospinal fluid and white matter were regressed out. Additionally, all functional images were normalized to Montreal Neurological Institute (MNI) space by Dartel and each voxel was resampled to 3 x 3 x 3 mm^3^. After normalization, images were spatial smoothed to a Gaussian kernel of 4 mm^3^, and performed linear trend removal and temporal band-pass filtering (0.01-0.08 Hz) for reducing the effects of low- and high-frequency noise. Finally, considering the potential impact of head motion, any participants with more than 1.5° angular motion and 1.5 mm maximum displacement were excluded. We also computed the mean frame-wise displacement (FD) of each participant which applied as a covariate in the further analysis (31).

To investigate whether the patterns of cerebellar-cerebral functionally-connected networks were different between MA and HC groups, seed-based functional connectivity analyses were carried out by selecting regions of interest (ROIs) in the cerebellum. These specific seeds have been documented to involve in cerebellar-cerebral FC networks such as executive control, default-mode, affective-limbic and sensorimotor networks, and were shown by group here (**Table 1**) (19, 26, 32–34). An average time series was extracted from each spherical ROI round the central point (6-mm radium). For each participant, correlation coefficients were computed between each seed and the voxels in the whole brain, which then converted to z-values using Fisher’s r-to-z transformation to improve the normality.

**Table 1:** Cerebellar seeds and Montreal Neurological Institute (MNI) coordinates grouped by network.

Cerebellar network	Cerebellar seed	Code	MNI (x, y, z)
Executive control network	L Crus I_ECN1_	ROI1	-12, -78, -28
	R Crus I_ECN1_	ROI2	12, -78, -28
	L Crus II_ECN2_	ROI3	-36, -70, -46
	R Crus II_ECN2_	RIO4	36, -68, -44
	L Lobule VI_ECN3_	ROI5	-36, -52, -34
	R Lobule VI_ECN3_	ROI6	36, -52, -34
Default-mode network	L Crus I_DMN_	ROI7	-32, -76, -34
	R Crus I_DMN_	ROI8	34, -80, -36
Affective-limbic network	R Lobule VI_LN_	ROI9	26, -64, -34
	L Lobule VI_LN_	ROI10	-26, -64, -34
	L Vermis_LN_	ROI11	-4, -80, -34
Sensorimotor network	R Lobule V_SMN_	ROI12	22, -52, -22
	L Lobule V_SMN_	ROI13	-20, -50, -24

L, left; R, right; ECN, executive control network; DMN, default-mode network; LN, affective-limbic network; SMN, sensorimotor network.

### Statistical Analysis

Differences in demographic data between MA and HC groups were compared using two-sample t-tests and chi-square tests. To identify the general pattern of cerebellar-cerebral FC maps and to examine the possible differences of cerebellar connection topologies in MA group, one-sample t-tests were conducted to demonstrate the spatial distribution of cerebellar-cerebral FC in two groups, respectively. Then, to determine the significant differences in FC maps between MA group and HCs, two-sample t-test was performed. Head motion and demographic variables including age, gender, and education were used as covariates in all functional connectivity analyses. Cerebral mask was used as an explicit mask generated by WFU_PickAtlas software (35, 36). The statistical threshold was set at corrected p < 0.05 (combination of individual voxel p < 0.001 and a minimum cluster size of 31), which determined by multiple comparisons with the AlphaSim correction (https://afni.nimh.nih.gov/afni/doc/manual/AlphaSim.pdf). Cohen’d was calculated to measure robustness of the findings, which was considered as large effect sizes with d value above 0.8 (37). Finally, for each region showing significantly different cerebellar-cerebral FC across the groups, the mean z-values were extracted and correlated against each clinical measurement (age of first MA-use, craving scores, addiction severity, etc.) of MA dependent subjects, after controlling for age, gender, and education. The significance level was set at p < 0.05, corrected for Bonferroni correction.

## Results

### Demographic and Clinical Characteristics

Data from five participants (3 MA patients and 2 HCs) were excluded for further analysis because of excessive head movement. The final analysis therefore included 34 MA patients and 31 HCs. Demographic characteristics and drug use information are shown in **Table 2**. Overall, the MA dependent subjects had a prolonged abstinence period from the last-time MA use to MRI scans for about six (5.85 ± 0.60) months. There was no group difference in terms of age, gender, years of education and mean displacement of head motion.

**Table 2:** Demographic and clinical characteristics of the participants.

	MUD group (n = 34)	HC group (n = 31)	t / χ^2^	P
Age (years)	32.15 ± 6.85	34.48 ± 7.73	-1.29	0.201
Gender (male / female)	18 / 16	21 / 10	1.48	0.224
Education (years)	9.76 ± 1.44	9.74 ± 2.16	0.05	0.960
Mean FD	0.09 ± 0.04	0.08 ± 0.04	0.91	0.364
Age of first MA-use (years)	23.74 ± 8.11	—	—	—
Duration of MA use (years)	6.59 ± 3.68	—	—	—
Dose of MA use per day (g)	0.51 ± 0.33	—	—	—
Frequency				
Every day	20 (58.8%)	—	—	—
3-5 times a week	10 (29.4%)	—	—	—
Once a week	4 (11.8%)	—	—	—
Abstinence (months)	5.85 ± 0.60	—	—	—
Craving (VAS)	4.89 ± 3.34	—	—	—
Addiction severity (ASI)	1.04 ± 0.27	—	—	—
Drug use severity	0.13 ± 0.09			

FD, frame-wise displacement; VAS, Visual Analogue Scale; ASI, Addiction Severity Index.

### Cerebellar-Cerebral Functional Connectivity in HC and MA Groups

**Figure 1:**
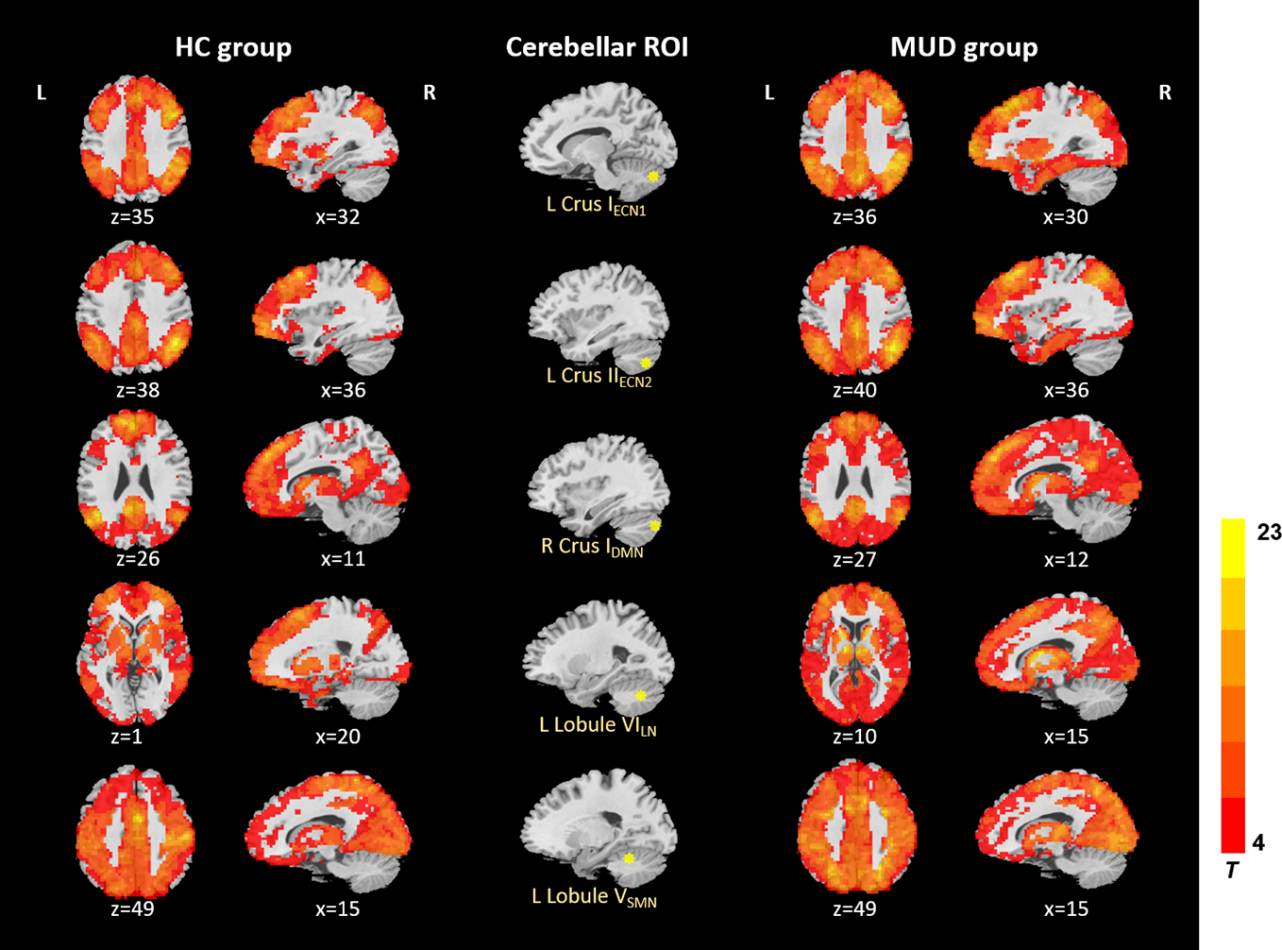
Resting-state cerebellar-cerebral functional connectivity in the HC group vs. MUD group (p < 0.001). ECN, executive control network; DMN, default-mode network; LN, affective-limbic network; SMN, sensorimotor network.

**Figure 2:**
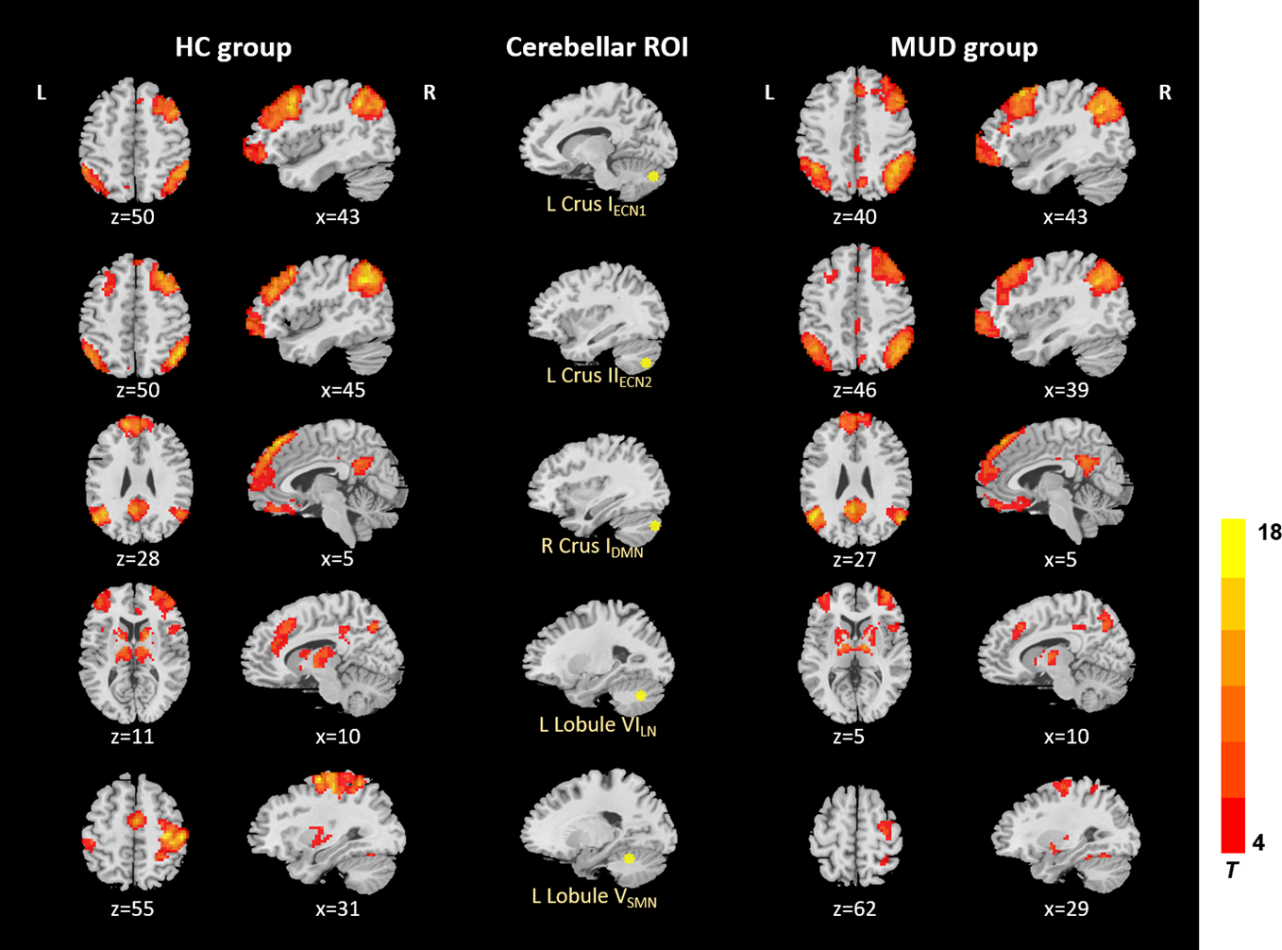
Resting-state cerebellar-cerebral functional connectivity in the HC group vs. MUD group (p < 0.0001). ECN, executive control network; DMN, default-mode network; LN, affective-limbic network; SMN, sensorimotor network.

In the HC and MA groups, the seeds of cerebellum both exhibited distributed FC with cerebrum (**Figure 1**). When we applied a more rigorous threshold of single voxel p < 0.0001 combined with Alphasim corrected p < 0.05, the seeds showed good specificity in cerebellar-cerebral FC networks (**Figure 2**) (32, 33), which was in agreement with previous researches. In general, the significant connectivity did occurr in each ROI with corresponding cerebral networks (19, 26). More specifically, the cerebellar seed regions in executive network exhibited connectivity mainly with superior frontal gyrus, inferior parietal gyrus and inferior temporal gyrus. The seed regions in default-mode network exhibited connectivity mainly with mPFC, PCC and precuneus. The seed regions in affective-limbic network exhibited connectivity mainly with thalamus, insula and caudate. The seed regions in sensorimotor network exhibited connectivity mainly with precentral gyrus, postcentral gyrus and supplementary motor area (**Figure 2**). Moreover, similar patterns of cerebellar-cerebral connected networks appeared across MA and HC groups.

### Between-Group Differences in Functional Connectivity

**Figure 3:**
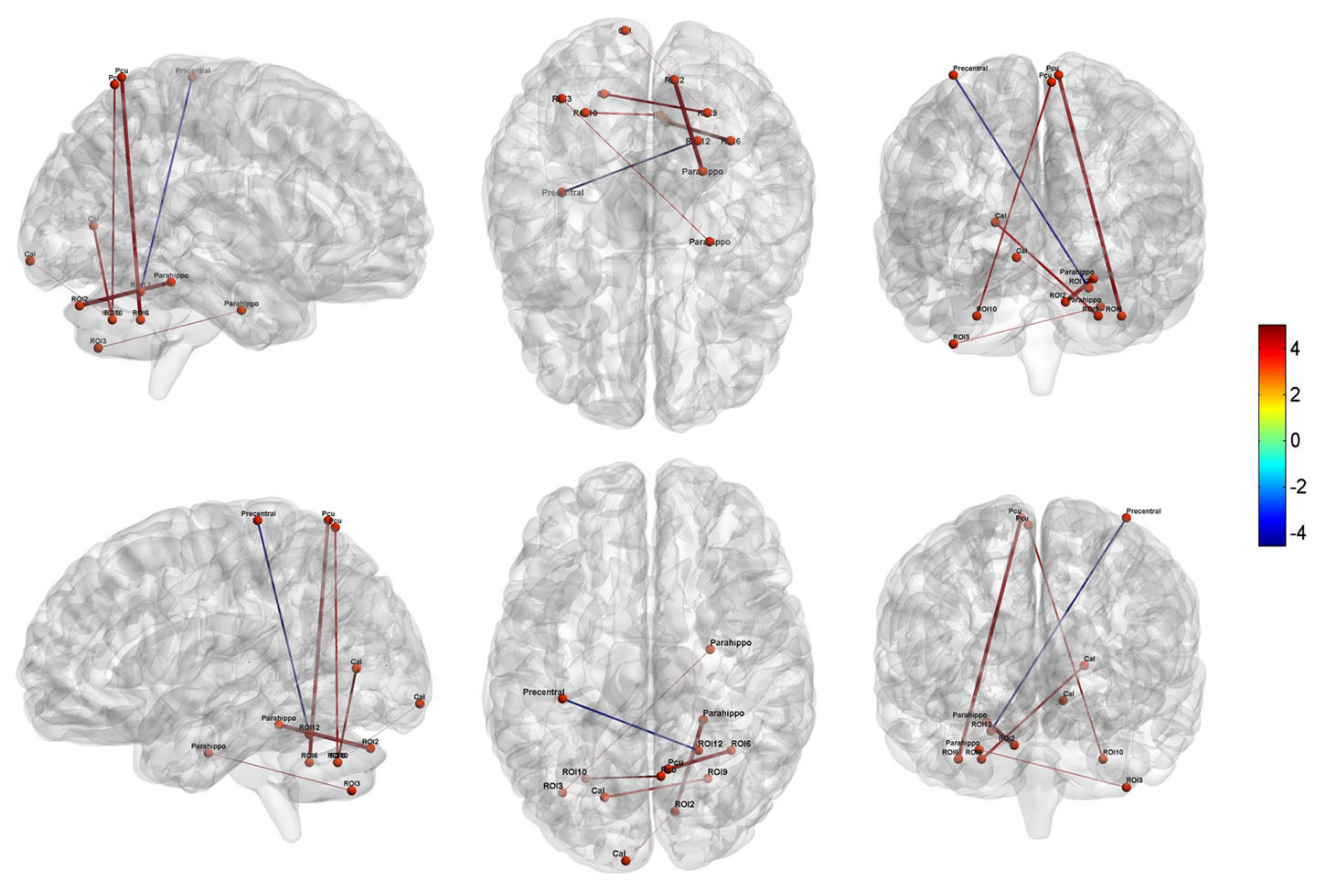
Altered cerebellar-cerebral functional connectivity in MA dependent individuals relative to HCs (P < 0.05, AlphaSim corrected). Parahippo, Parahippocampal; Pcu, Precuneus; Cal, Calcarine.

Compared to the controls, MA dependent individuals showed significantly altered cerebellar connectivity with several cortical and subcortical areas, primarily located in default-mode, affective-limbic and sensorimotor networks (**Table 3** and **Figure 3**). For the seeds of executive network in cerebellum, the patients exhibited higher FCs between right Crus I_ECN1_ and right parahippocampal gyrus, between left Crus II_ECN2_ and right parahippocampal gyrus, and between right Lobule VI_ECN3_ and right precuneus. For the seeds of affective-limbic network, the patients exhibited higher FCs between right Lobule VI_LN_ and left calcarine and between left Lobule VI_LN_ and right precuneus. For the seeds of sensorimotor network, the patients exhibited lower FCs between right Lobule V_SMN_ and left precentral gyrus.

**Table 3:** Altered cerebellar-cerebral FC in the MA group relative to HC group.

Cerebellar seeds	Brain regions	Voxel	MNI (x, y, z)	T values	Cohen’s d
MUD > HC					
Executive control network					
R Crus I_ECN1_	R Parahippocampal gyrus	79	24, -39, -18	5.0328	1.32
L Crus II_ECN2_	R Parahippocampal gyrus	32	27, -9, -30	4.2047	1.22
R Lobule VI_ECN3_	R Precuneus	40	9, -60, 69	4.9231	1.07
Affective-limbic network					
R Lobule VI_LN_	L Calcarine	53	-9, -99, -9	4.1318	0.91
	L Calcarine	162	-18, -72, 6	4.6142	0.92
L Lobule VI_LN_	R Precuneus	43	6, -63, 66	4.5102	1.00
					
MUD < HC					
Sensorimotor network					
R Lobule V_SMN_	L Precentral gyrus	31	-36, -30, 69	-4.5384	1.17

L, left; R, right; ECN, executive control network; LN, affective-limbic network; DMN, default-mode network; corrected p < 0.05, corrected for Alphasim correction.

### Correlation Between Cerebellar-Cerebral FCs and Clinical Characteristics of MA Group

**Figure 4:**
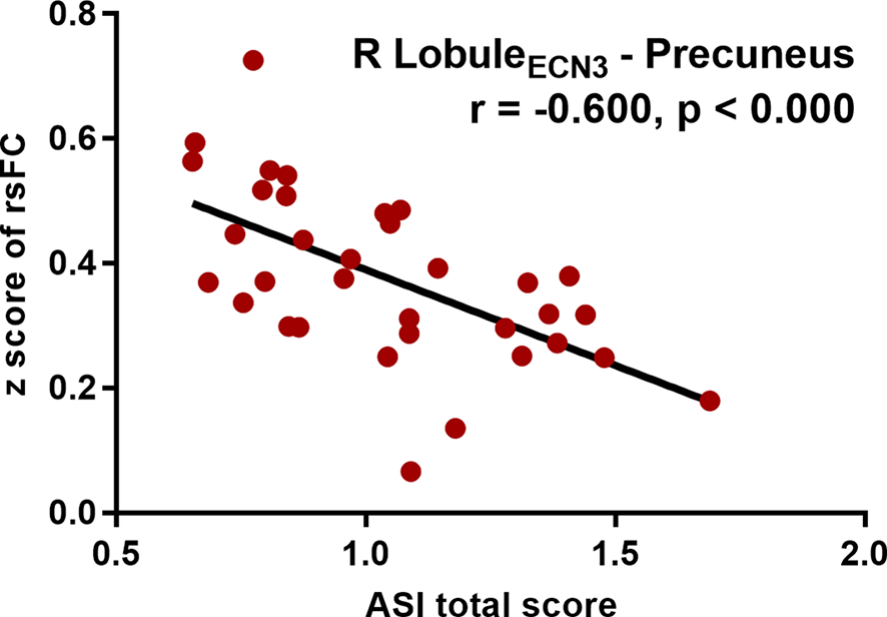
Partial correlation between the severity of drug addiction and right Lobule_ECN3_ – Precuneus functional connectivity, controlling for age, gender and education (P < 0.05, Bonferroni corrected). ASI, Addiction Severity Index; rsFC, resting-state functional connectivity; R, right; ECN, executive control network.

Within the MA group, the addiction severity was negatively correlated with strength of the FC between right Lobule VI_ECN3_ and precuneus (r = -0.600, p < 0.000 uncorrected, p = 0.008 Bonferroni corrected) (**Figure 4**). But beyond that, there was no significant correlation between the altered cerebellar-cerebral FCs and other clinical characteristics.

## Discussion

In the present study, we systematically explored resting-state cerebellar-cerebral functional connectivity in MA dependent individuals with long-term abstinence. Using seed-based FC analysis, we identified the organization of cerebellar-cerebral functional networks in both groups, which is consistent with previous findings of the functional topography of cerebellum and its role in cognitive and affective processing (19, 26). Compared to healthy controls, the patients exhibited altered FCs between cerebellum and several cerebral functional networks, including the affective-limbic, default-mode, and sensorimotor networks. Additionally, there was a negative correlation of the addiction severity with FC strength between right cerebellar lobule VI_ECN3_ and precuneus in MA group. Overall, it is hoped that the current results may contribute to the consideration of cerebellum as part of pathophysiological circuitry of MA use disorder.

### General Patterns of Cerebellar-Cerebral FC in Long-Term Abstinent MA Patients Compared to HCs

In general, current studies found that intrinsic FCs between cerebellum and cerebrum were significantly higher in the long-term abstinent MA patients for brain areas of default-mode and affective-limbic networks. There was only a small cluster in the sensorimotor network showing lower connectivity. Unexpectedly, these overall findings were somewhat incongruent with previous addiction literatures with respect to cerebellar-cerebral connectivity (38, 39). A fMRI study in prescription opioid-dependent patients reported a reduction in FCs strength between the cerebellum and limbic networks (38). Relative to matched controls, opioid dependent patients showed decreased cerebellar connectivity with insula, amygdala and nucleus accumbens (NAc) (38). Considering that the participants in our study were under a long-term abstinence periods, this may serve the reason why large overconnectivity predominated. In fact, a recent review has put forward the theory supporting cerebellum’s role as a modulator in addiction disease (22). In the model for cerebellar function in addiction, the researchers propose that cerebellum may play an important role in maintaining balance of the brain circuits underlying cognitive and affective processing (22). Moreover, our group has revealed that the MA dependent individuals gained larger volume in cerebellar gray matter as abstinence periods prolonged, which demonstrates that the overconnectivity in resting-state FC may be a pattern of regulation of homeostatic balance through cerebellar-cerebral functional networks (23).

Our results were also beyond expectation regarding the inconformity between the seeds of specific networks in cerebellum and altered brain areas in cerebrum. For example, in MA group, both seeds in executive control and affective-limbic networks showed significantly higher connectivity with precunues, which is ordinarily considered as a key node of DMN (40). As been illustrated by animal studies, repetitive drug administration could induce activity-dependent plasticity alteration in cerebellar synapses that may lead to rewiring of cerebellar microstructures and reorganization of functional networks (21, 41–43). Resting-state networks normally represent coherent signal fluctuations across different brain regions that share the common function (44). Thus, the phenomenon of inconformity possibly reflects that functional reorganization may happen in cerebellar-cerebral functional networks during the abstinence periods in MA users. However, further longitudinal studies are required to confirm these findings in different addiction-related processes.

### Dysconnectivity of Cerebellum-Cerebral Functional Networks in MA Group

In the MA group, we found enhanced FC of the cerebellum with precuneus, a core component of posterior DMN (40). The precuneus has been supposed to play a role in processing self-directed thoughts, monitoring internal states and evaluating emotional valence of personal events (45, 46). Previous studies have consistently reported the functional abnormality of this structure and its relation to clinical symptoms of strong reaction to drug-related cues in people with addiction problems (47, 48). For example, in cocaine abusers, Tomasi et al. found that drug-associated stimuli could elicit altered activity in precuneus, which reflects its involvement in compromised process of drug-related cue reactivity (49). On the other hand, studies have also demonstrated cerebellar activation by presenting drug-related cues (24, 50). So, the present finding of higher cerebellar-precuneus FC suggests that this abnormal connectivity may underlie drug-cues directed attention and ruminatory behavior, which lead to cue-elicited craving and drug consumption in MA users. Furthermore, when compared to non-relapsers, the heroin relapsers showed altered activity in both precuneus and cerebellum when drug-related cues were encountered (51, 52). Combined with our results, the aberrant cerebellar-precuneus pathway might provide a promising neural target for predicting the risk of relapse in MA use individuals. In addition, significant negative correlation was detected between right cerebellar lobule VI_ECN3_-precuneus and the addiction severity in MA group. As previous studies have proposed, the cerebellum, which has extensive connectivity with many cerebral regions including precuneus, acts like a “modulator” role in regulating drastically altered neural circuits in the addicted brain (22). In addiction disease, inefficient cerebellar-cerebral communication would result in impaired reward/salience processing and inhibitory control (22, 25). Hence, our result of cerebellar-precuneus coupling negative correlated with addiction severity level reflects that heavy MA use may hinder the cerebellar ability to regulate internal condition of drug craving that precuneus mainly involve, especially when salient drug-related cues are detected. We hypothesized that cerebellar-precuneus loop may also be trait alteration of MA use and neurobiological biomarker for drug-related cue reactivity in MA dependent subjects.

Besides, overconnectivity between the cerebellum and affective-limbic network, such as parahippocampus, had also been discovered. The parahippocampus has been reported to be implicated in drug-related cue memories *via* its role in Pavlovian associative learning (53). Remarkably, evidence arising over the recent years has shown that cerebellum also mediates storage and consolidation of drug-dependent Pavlovian memories (25). A neuroimaging study on healthy volunteers indicated that self-referential pictures could alter the activation of cerebellum and parahippocampus, coincided with other brain areas related to motivational processing (54). Together with these findings, our results imply that disrupted cerebellar-parahippocampal FC may play some important role in drug-related conditioned memories, which could modulate the motivational drive for drug use and promote drug intake triggered by drug-associated stimuli. From a clinical perspective, this higher interaction may suggest that abstinent MA patients still had difficulties in preventing drug-related cue induced craving, which lead to an enhanced regulation of affective-limbic network by cerebellum to stabilize motivational system in MA users.

We also found aberrant FCs between the cerebellum and sensorimotor network in MA group. Firstly, the connectivity of cerebellum with calcarine (visual cortex) was enhanced in MA dependent subjects, which was congruent with prior studies regarding the function of cerebellum in visual processing (5). Our finding was also in support of the deficits in attention and visuo-spatial abilities in MA patients with long-term abstinence. Additionally, we also detected lower FC between the cerebellum and precentral gyri, which is the key node of motor network. The cerebellum and precentral area both involve in automatized and habitual behaviors (55, 56). With repeated drug administration, a sequence of drug-seeking actions becomes habitual and automatized, that constitutes the core process of transition to addiction (53). Using cue reactivity paradigm, previous findings have found cue-induced activations in the cerebellum and motor cortex (57, 58), and that the brain activations were associated with the degree of automatized responses toward drug-related cues (57). Given these findings, we hypothesized that abnormal FC of the cerebellar-precentral gyri coupling could be the neural basis of habitual drug-seeking behavior and compulsive drug use in MA use disorder. Moreover, this lower FC also raises the possibility that compulsivity is a general characteristic of drug dependence even when patients were under prolonged abstinence periods. And impaired cerebellar-motor network interactions may be involved in MA addiction *via* their failure to control the habitual drug consumption, which could be a potential target to prevent from developing compulsive drug abuse in the future.

In recent years, increasing researches have come to investigate extensively the dysfunction of cerebellum in addictive behaviors (21, 22, 24, 25). Our findings, which were consistent with many previous studies, revealed that MA dependent individuals also demonstrated functional impairments in cerebellum, especially the intrinsic cerebellar-cerebral functional networks. Interestingly, a few investigations have shown that some impaired clinical properties in people with substance use disorders could be recovered by treatment for drug use, along with cerebellar function normalized (59–61). For example, Froeliger et al. identified that cerebellar-NAc FC was a biomarker for outcome of self-reported craving after N-Acetylcysteine administration in non-treatment smokers (61). However, despite these achievements, the study of clinical mechanism of cerebellar-cerebral FC in the therapy of patients with addiction problems is still in its infancy, that further works are required to clarify cerebellar contribution to the treatment of MA addiction.

There are several limitations of our study that should be acknowledged. First, our sample size was relatively limited. Thus, a larger sample study should be employed in the future to replicate the current results. Second, the study was cross-sectional in design, and all MA dependent individuals were under long-term abstinent periods. It is not appropriate to determine the causality between disrupted cerebellar-cerebral FC and developing MA characteristics. Therefore, further longitudinal investigations with a long-time window, like periods from drug using to short-term abstinence, are required. Third, the altered cerebellar-cerebral FC mostly supported the content of drug-related cues processing, but we failed to find a relationship between craving scores and the significantly aberrant connectivity. One fMRI study showed that drug-induced craving was positively correlated with activity in the cerebellar vermis (62). In our study, we only assessed the craving in a natural state, so further studies should also evaluate the cue-elicited craving and its correlation with cerebellar-cerebral connectivity. Lastly, we failed to detect any differences in functional connectivity between cerebellum and reward-related areas, like striatum. One of the main reasons was that the relatively small sample size may reduce statistical power in the present neuroimaging study. For another reason, the whole-brain voxel-wise analysis used here increased the number of multiple comparisons that these reward-processing brain areas were hard to survive due to small volume of few voxels. Hence, further works with a more specific hypothesis are needed to explore the connectivity of cerebellum with reward-related areas in larger sample size.

## Conclusion

In summary, this study directly revealed the cerebellar-cerebral dysconnectivity in MA dependent individuals with long-term abstinence. Specifically, MA patients showed disrupted FC between the cerebellum and several cerebral functional networks, including the DMN, affective-limbic and sensorimotor networks, which mainly engaged in processing drug-related cues. This study also found that the FC of right cerebellar lobule VI- precuneus coupling was correlated to addiction severity, which implied a trait alteration of MA use. Overall, this was the first investigation to systematically explore resting-state cerebellar-cerebral FC in MA use disorder. We hope that the present results will be helpful to involve cerebellum in neurobiological mechanism and treatment target of MA addiction in the future.

## Data Availability Statement

The datasets generated for this study are available on request to the corresponding authors.

## Ethics Statement

The studies involving human participants were reviewed and approved by Ethics committee of Shanghai Mental Health Center. The patients/participants provided their written informed consent to participate in this study.

## Author Contributions

MZ and HJ were responsible for the study concept and design. XL and HS conducted the data analysis and drafted the manuscript. NZ and JD helped design the study. TC helped acquire the clinical and imaging data. KX, DX, and WS helped guarantee the study. All authors provided critical revision of the manuscript for important intellectual content. All authors critically reviewed content and approved final version for publication.

## Funding

This work was supported by the National Key R&D Program of China (2017YFC1310400), National Nature Science Foundation (81771436, 81801319, 81601164), Program of Shanghai Academic Research Leader (17XD1403300), Shanghai Municipal Health and Family Planning Commission (2017ZZ02021), Municipal Human Resources Development Program for Outstanding Young Talents in Medical and Health Sciences in Shanghai (2017YQ013), Program of Science and Technology Innovation Plan in Shanghai (18411961200), Shanghai Key Laboratory of Psychotic Disorders (13DZ2260500), Shanghai Municipal Science and Technology Major Project (2018SHZDZX05), and Shanghai Clinical Research Center for Mental Health (19MC1911100). The funders have no role in study design, data collection and analysis, decision to publish, or preparation of the manuscript.

## Conflict of Interest

The authors declare that the research was conducted in the absence of any commercial or financial relationships that could be construed as a potential conflict of interest.
